# Complete Genome Sequences of Rhodothermus marinus Strains AA2-13 and AA3-38, Isolated from Arima Onsen Hot Spring in Japan

**DOI:** 10.1128/MRA.01475-19

**Published:** 2020-01-09

**Authors:** Natsuki Tomariguchi, Kentaro Miyazaki

**Affiliations:** a Department of Life Science and Biotechnology, Bioproduction Research Institute, National Institute of Advanced Industrial Science and Technology, Tsukuba, Ibaraki, Japan; b Faculty of Life Sciences, Toyo University, Itakura, Gunma, Japan; c Department of Computational Biology and Medical Sciences, Graduate School of Frontier Sciences, The University of Tokyo, Kashiwa, Chiba, Japan; Georgia Institute of Technology

## Abstract

We isolated Rhodothermus marinus strains AA2-13 and AA3-38 from Arima Onsen, a hot spring in Japan, and sequenced their genomes. The average nucleotide identity between their genomes was 99.2%, and that with the genome of R. marinus strain DSM 4252^T^ (isolated from Iceland) was ∼95.2%, suggesting close relationships among these strains.

## ANNOUNCEMENT

The *Rhodothermus* genus belongs to the phylum *Bacteroidetes* and has been found in geothermal habitats. *Rhodothermus* has gained much attention as a source of industrial enzymes (1) and as a target for protein engineering (2) because of its thermostability. Since the discovery of Rhodothermus marinus DSM 4252 (the type strain of R. marinus) from the Blue Lagoon geothermal spa in Iceland (3), several *Rhodothermus* species have been isolated from high-temperature environments (4, 5).

We isolated thermophilic and halophilic R. marinus strains from the nonvolcanic, oceanic Arima Onsen (a hot spring) in Kobe, Japan, on 20 March 2017 (6). The environmental sample was spread over marine agar plates (Difco) and incubated at 65°C. After 48 h, dark-pink colonies that appeared on the plates were examined by sequencing of the full length of the 16S rRNA gene, which was amplified using the primers Thermus_1F and Thermus_1521R (7). Many of the colonies were associated with Thermus thermophilus (8), one with Rubrobacter xylanophilus (9), and the others with R. marinus (∼99% identity with the R. marinus DSM 4252^T^ 16S rRNA gene [GenBank accession number NR_029282.2]). Among the R. marinus strains, two strains, AA2-13 and AA3-38, were subjected to genome analysis. The strains were grown in marine broth (Difco) at 65°C for 24 h. Genomic DNA was purified using the 1^-Step^ DNA isolation kit for bacteria (nexttec Biotechnologie), according to the manufacturer’s instructions.

Genome analysis was conducted by employing a combination of Illumina short-read and Oxford Nanopore Technologies (ONT) long-read sequencing. For short-read sequencing, genomic DNA was sheared to 100-bp fragments, adaptor ligated, and amplified to generate DNA libraries using the Nextera DNA library preparation kit (Illumina). The libraries were subjected to paired-end sequencing (2 × 100 bases) on the Illumina HiSeq 2500 platform. A total of 8,154,381 reads (815 Mb) and 7,894,203 reads (789 Mb) were produced for AA2-13 and AA3-38, respectively. Adapters and low-quality data were trimmed using fastp v.0.19.5 (10). Default parameter settings were applied for software analyses throughout this study.

For long-read sequencing, short genomic fragments were removed using a short-read eliminator kit (Circulomics). Sequencing was performed using a GridION X5 sequencer (ONT). Unfragmented genomic DNA (1 μg) was used for library construction with a ligation sequencing kit (ONT). The library was then analyzed in a FLO-MIN106 R9.41 flow cell (ONT) for 12 h. Base calling was conducted using Guppy v.3.0.3 to generate 140,887 reads (2.09 Gb) and 467,442 reads (2.87 Gb), with average lengths of 14,800 bases (longest read, 269,105 bases; *N*_50_, 37,152 bases) and 6,143 bases (longest read, 156,140 bases; *N*_50_, 20,011 bases) for AA2-13 and AA3-38, respectively. The raw read data were filtered (average Phred quality values, >8.0) using NanoFilt v.2.3.0 (11).

The hybrid assembly of long- and short-read data was conducted using Unicycler v.0.4.4 (12), followed by final polishing with Pilon v.1.23 (13). For both strains, a single circular chromosome was generated. Unlike in DSM 4252^T^, no plasmids were found in AA2-13 or AA3-38. Automated annotation was performed using DFAST v.1.1.0 (14). The features of the genomes are presented in Table 1. Average nucleotide identity (ANI) analysis was carried out using the Web-based OrthoANIu calculator (15), which revealed that the genome sequences of AA2-13 and AA3-38 were nearly identical (99.2% ANI), with no large gaps or rearrangements. As of 28 November 2019, two complete genome sequences had been determined for R. marinus (strains DSM 4252^T^ [GenBank accession number NC_013501.1] and SG0.5JP17-172 [GenBank accession number NC_015966.1]). Despite the distant habitats, strains AA2-13 and AA3-38 showed gene arrangements similar to that of strain DSM 4252^T^, with ANIs of ∼95.2%, suggesting close relationships among these strains.

**TABLE 1:** Genome statistics and features of R. marinus isolates

Strain	Chromosome or plasmid	Length (bp)	GC content (%)	No. of coding sequences	No. of rRNAs	No. of tRNAs	GenBank accession no.
AA2-13	Chromosome	3,440,105	64.1	2,981	3	45	AP019796
AA3-38	Chromosome	3,430,829	64.1	2,980	3	46	AP019797
DSM 4252^T^	Chromosome	3,261,604	64.5	2,797	3	45	NC_013501.1
	Plasmid (pRMAR01)	125,133	58.2	107	0	0	NC_013502.1

### Data availability.

The GenBank accession numbers for the complete genome sequences of R. marinus strains AA2-13 and AA3-38 are AP019796 and AP019797, respectively (Table 1). Raw sequencing data were deposited in the DDBJ Sequence Read Archive under accession number DRA008620 (GenBank BioProject number PRJDB8496 and BioSample accession numbers SAMD00177810 [strain AA2-13] and SAMD00177811 [strain AA3-38]).
